# Bridging Global Disparities in Drug Allergy Through AI‐Assisted Training for Non‐Specialists: Findings From the Multinational ADAPT‐2 Course

**DOI:** 10.1111/cea.70213

**Published:** 2026-01-20

**Authors:** Hugo W. F. Mak, Juan Meng, Freya K. L. Chung, Jasmine T. Y. Lee, Inoka Sepali Aberathna, Uvini Amarasekara, Thushali N. D. Ranasinghe, Jeewantha Jayamali, Qintai Yang, Min Zhou, Liang Chen, Jin‐Xian Huang, Weihong Shi, Rishabh Kulkarni, Jonny Grant Peter, Jason A. Trubiano, Kamal Chandima Jeewandara, Michaela Lucas, Philip H. Li

**Affiliations:** ^1^ Division of Rheumatology and Clinical Immunology, Department of Medicine Queen Mary Hospital, The University of Hong Kong Hong Kong SAR China; ^2^ Department of Allergy and Immunology West China Hospital, Sichuan University Chengdu China; ^3^ Allergy Immunology and Cell Biology Unit, Department of Immunology and Molecular Medicine University of Sri Jayewardenepura Nugegoda Sri Lanka; ^4^ Department of Allergy The Third Affiliated Hospital of Sun Yat‐Sen University Guangzhou Guangdong China; ^5^ Division of Rheumatology & Clinical Immunology, Department of Medicine University of Hong Kong‐Shenzhen Hospital Shenzhen Guangdong China; ^6^ Medical School University of Western Australia Nedlands Western Australia Australia; ^7^ Department of Immunology SCGH and PCH Nedlands Western Australia Australia; ^8^ Division of Allergy and Clinical Immunology, Department of Medicine, Groote Schuur Hospital University of Cape Town Cape Town South Africa; ^9^ Allergy and Immunology Unit University of Cape Town Lung Institute Cape Town South Africa; ^10^ Department of Infectious Diseases, Centre for Antibiotic Allergy and Research Austin Health Heidelberg Victoria Australia; ^11^ National Centre for Infections in Cancer Peter MacCallum Cancer Centre Melbourne Victoria Australia; ^12^ Department of Infectious Diseases University of Melbourne at the Peter Doherty Institute for Infection and Immunity Melbourne Victoria Australia; ^13^ Department of Immunology Pathwest Laboratory Medicine Nedlands Western Australia Australia

**Keywords:** artificial intelligence, disparities, drug allergy, education, non‐allergist

## Abstract

**Background:**

Mislabelled drug allergy (DA) remains a global public health challenge. A prior randomised trial (ADAPT) demonstrated that an intensive educational course improved DA knowledge and confidence among non‐specialists. However, ADAPT was restricted to English‐speaking participants and its generalisability remains unknown. To address this, a multinational implementation study expanding ADAPT (ADAPT‐2) was performed.

**Methods:**

Non‐allergist physicians from Colombo (Sri Lanka), Guangzhou and Shenzhen (Mainland China), Hong Kong (Special Administrative Region of China) and Perth (Australia) completed a standardised DA educational course. In Mainland China, training was delivered via AI‐assisted video localisation (converted into Mandarin while preserving the speaker's voice with lip‐synced adaptation). DA knowledge, confidence and practice were assessed before and after completion. Subgroup analyses compared pre–post changes between Advanced Economies (AE: Australia, Hong Kong) and Emerging Economies (EE: Mainland China, Sri Lanka).

**Results:**

Of 181 participants, overall baseline knowledge (53.5% ± 17.2%) and confidence (47.5% ± 22.7%) scores were suboptimal. EE participants had a lower knowledge level than AE (49.1% ± 15.5% vs. 70.1% ± 12.7%; *p <* 0.001). Following ADAPT‐2, both knowledge (72.5% ± 16.0%, *p* < 0.001) and confidence (71.3% ± 17.5%, *p* < 0.001) scores significantly improved across all groups. ADAPT‐2 delivered by AI‐assisted video localisation was non‐inferior to the English course in effectiveness (*p* > 0.05) and achieved high participant satisfaction (98.9% as ‘somewhat clear’ or better in clarity).

**Conclusions:**

Deficits in DA knowledge persist widely among non‐specialists, with marked disparities between AE and EE. ADAPT‐2 bridged these gaps by universal improvements in both DA knowledge and confidence. AI‐assisted training represents a scalable, equitable strategy for global implementation of standardised and evidence‐based DA education.

**Trial Registration:**

ADAPT: NCT06399601

## Graphical abstract

The multinational ADAPT‐2 initiative conducted in Australia, Hong Kong, mainland China and Sri Lanka demonstrated that AI‐assisted training is effective in addressing the significant disparities between advanced and emerging economies in non‐specialist physicians' drug allergy knowledge and confidence.

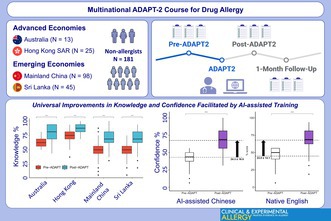

## 1 Introduction

Mislabelled drug allergy (DA), in particular to antibiotics, has emerged as a critical public health concern and a major threat to global antimicrobial stewardship [1, 2]. Although approximately 10%–20% of the general population is labelled allergic to at least one drug, especially to β‐lactams (BL)—up to 90% of these labels are found to be incorrect upon formal evaluation [3, 4, 5, 6, 7, 8]. Mislabelled DA creates barriers to appropriate prescribing and leads to unnecessary medication avoidance, resulting in a myriad of adverse consequences, including increased antimicrobial resistance, suboptimal clinical outcomes and diminished health‐related quality of life [9, 10, 11, 12].

Preventing mislabelling and removing incorrect drug allergy labels (‘delabelling’) are essential to tackling this pervasive public health challenge [13, 14, 15, 16, 17]. However, in many non‐Western countries, especially in the Asia‐Pacific (APAC), access to specialist allergy services and formal allergy training remains severely limited [18, 19, 20]. Fortunately, a growing body of evidence indicates that the majority of DA labels, especially those related to penicillins, are classified as ‘low risk’ and can be safely evaluated by non‐allergist physicians or trained allied health professionals [21, 22, 23, 24, 25]. This paradigm shift is exemplified by regional clinical pathways, such as by the Asia Pacific Association of Allergy, Asthma and Clinical Immunology, which provides a risk‐stratification strategy that enables non‐specialists to perform delabelling in ‘low‐risk’ patients with penicillin allergy [26].

However, to enable effective multidisciplinary collaboration to tackle the global pandemic of mislabelled DA, it is essential to ensure that a diverse range of clinicians (both allergists and non‐allergists) are thoroughly trained to evaluate/manage suspected DA with confidence and competence [27]. Unfortunately, standardised and evidence‐based DA education courses tailored for non‐specialists remain scarce, especially in low‐ and middle‐income regions where accredited allergy training pathways are lacking [28]. A further critical barrier is language: while English dominates international guidelines and research literature, it is neither the primary language nor the medium of instruction in medical education across much of the APAC. In recent years, artificial intelligence (AI) has advanced rapidly in natural language processing and machine translation, offering enormous potential to overcome this language barrier [29, 30]. Notably, AI‐assisted video localisation technologies can dynamically translate instructional content into local languages while preserving the original speaker's voice and synchronising lip movements with the translated speech. These innovations hold promise for significantly improving comprehension, retention and engagement among non‐English‐speaking learners.

A randomised crossover trial, Advancing Drug Allergy & Penicillin Testing (ADAPT), previously demonstrated that an intensive educational course improved DA knowledge and confidence among non‐specialists [31]. However, ADAPT was limited to English‐speaking participants, and its generalisability to other populations remains unknown. To address this, we conducted ADAPT2, a multinational implementation study expanding the ADAPT curriculum across four APAC regions with distinct economic and healthcare systems: Colombo (Sri Lanka), Guangzhou and Shenzhen (Mainland China), Hong Kong (Special Administrative Region of China) and Perth (Australia). In Mainland China, the course was delivered using AI‐assisted video localisation, which translates instructional content into Mandarin Chinese while preserving the original speaker's voice and synchronising lip movements to match the translated audio. This study assesses the generalisability and effectiveness of ADAPT2 in improving DA knowledge, confidence and clinical practice among non‐specialist participants across diverse settings, as well as the utility of AI‐assisted translation in the delivery of DA education.

## 2 Methods

This study was an international, multicentre extension of the previously published ADAPT trial [31]. In brief, ADAPT was an intensive, 1‐day interventional education course designed and delivered in English by an international faculty of DA experts. The pilot study in Hong Kong demonstrated that it was effective for non‐specialists to improve both DA knowledge and confidence. In ADAPT‐2, we invited local experts from each participating locality to review, comment and approve the same educational course with reference to their own region‐specific contexts and needs. In addition, the framework and content of ADAPT‐2 were overseen and approved by an expert panel with members from different continents (Asia, Africa and Australia) and of different specialties (allergists, infectious disease physicians and otolaryngologists). The content and instructional faculty remained unchanged, and were implemented across all participating regions, except in Mainland China, where all educational materials were delivered in Chinese (Mandarin) via AI‐assisted training videos. These videos were generated using publicly available AI platforms, including Rask (Rask AI, www.rask.ai) and HeyGen (HeyGen, www.heygen.com), which translate and adapt English‐language videos into Chinese while preserving the original speaker voices and synchronising lip movements. All AI‐assisted content underwent rigorous manual review and correction by two independent, bilingual allergists fluent in both English and Chinese to ensure linguistic accuracy.

Regardless of delivery language, the training was uniformly administered in a hybrid format, combining pre‐recorded video lectures with standardised teaching materials. At least one faculty member (who must be local) was physically present at each site to supervise sessions and address participant queries. Full, in‐person attendance was mandatory for all participants, who were recruited through institutional public advertisements and affiliated social media channels. Eligibility was restricted to actively practicing physicians with proficiency in either English or Chinese (for Mainland China cohorts). Participant demographic and professional information were also collected (excluding those from Mainland China due to privacy restrictions). Participating regions included Colombo (Sri Lanka), Guangzhou and Shenzhen (Mainland China), Hong Kong (Special Administrative Region of China) and Perth (Australia). Regions were classified as either ‘Advanced Economies’ (AE) or ‘Emerging Economies’ (EE), based on the International Monetary Fund's country classification system as of 1/8/2024. The study protocol was approved by the Institutional Review Board of the University of Hong Kong/Hong Kong West Cluster, and all participants provided written informed consent.

ADAPT‐2 employed the same set of outcome measures as the original ADAPT trial, which consists of (1) a validated 16‐question knowledge assessment tool, (2) a questionnaire for participants' DA‐related practice and self‐reported management confidence, and (3) an evaluation survey, with three additional questions for participants from Mainland China regarding the clarity and understandability of the AI‐assisted translations. Details of the course and various outcome measures can be found in the original publication [31]. All participants completed the knowledge and practice questionnaires immediately before and after the training. A follow‐up practice survey was distributed at least 1 month post‐intervention to assess retention and implementation.

All statistical analyses except sample size calculation were performed using IBM SPSS Statistics, version 28.0 (IBM Corp., Armonk, NY, USA). Descriptive statistics summarised participant characteristics and survey responses. Categorical variables are presented as *n* (%), and continuous variables as mean ± standard deviation, unless otherwise specified. Between‐group comparisons (AE vs. EE; participants of different demographics and professional background) employed chi‐square tests for categorical variables and independent *t*‐tests for continuous variables. Within‐group pre‐post comparisons of knowledge and confidence scores used paired *t*‐tests for continuous outcomes and McNemar's test for binary/categorical outcomes. Sample size was calculated on R version 4.3.1 (R Foundation, Vienna, Austria) via the *pwr* package, where 44 subjects were needed to detect a change of moderate effect (Cohen's *d* = 0.5) at a statistical power of 90% and a two‐sided level of significance of 5%. Statistical significance was defined as a two‐tailed *p* value < 0.05.

## 3 Results

A total of 181 practicing non‐allergist physicians completed ADAPT‐2 and were included in the analysis: 13 (7.2%) from Australia, 25 (13.8%) from Hong Kong, 98 (54.1%) from Mainland China (recruited from Guangzhou and Shenzhen) and 45 (24.9%) from Sri Lanka. Overall, 47.0% were female, 72.3% practiced in the public sector and 39.8% had more than 10 years of clinical experience. Baseline characteristics of all participants are summarised in Tables 1 and 2.

**TABLE 1:** Demographic and professional background of participants.

	Total^a^	Advanced economies	Emerging economies^a^	*p*
	*N* = 83	*N* = 38	*N* = 45	
Sex				
Male	39 (47.0)	21 (55.3)	18 (60.0)	0.165
Female	44 (53.0)	17 (44.7)	27 (60.0)	
Years of clinical experience				
< 10 years	42 (50.6)	17 (44.7)	25 (55.6)	0.326
≥ 10 years	41 (49.4)	21 (55.3)	20 (44.4)	
Type of practice				
Private	23 (27.7)	16 (42.1)	7 (15.6)	0.007*
Public	60 (72.3)	22 (57.9)	38 (84.4)	
Specialty				
Internal medicine	28 (33.7)	14 (36.8)	14 (31.1)	0.077
Family medicine/General practitioner	27 (32.5)	12 (31.6)	15 (33.3)	
Paediatrics	9 (10.8)	7 (18.4)	2 (4.4)	
Other	19 (22.9)	5 (13.2)	14 (31.1)	

*Note:* *p < 0.05.

^a^ Data from Mainland China not available.

**TABLE 2:** Baseline characteristics of participants before ADAPT.

	Total	Advanced economies		Emerging economies		*p* ^a^
		Australia	Hong Kong	Mainland China	Sri Lanka	
	*N* = 181	*N* = 13	*N* = 25	*N* = 98	*N* = 45	
In your experience, approximately what proportion of patients have any drug allergy labels in their medical records?						
< 1%	50 (35.0)	2 (15.4)	1 (4.0)	41 (41.8)	9 (20.0)	< 0.001*
1%–5%	68 (47.6)	4 (30.8)	12 (48.0)	43 (43.9)	25 (55.6)	
6%–10%	17 (11.9)	3 (23.1)	9 (36.0)	9 (9.2)	8 (17.8)	
More than 10%	8 (5.6)	4 (30.8)	3 (12.0)	5 (5.1)	3 (6.7)	
In your experience, what are the top 3 most common drug allergy labels you have encountered?						
β‐lactams	225 (41.4)	13 (33.3)	30 (40.0)	129 (43.9)	53 (39.5)	N/A
Non‐steroidal anti‐inflammatory drugs	104 (19.2)	2 (5.1)	20 (26.7)	42 (14.3)	40 (29.6)	
Radiological contrasts	46 (8.5)	0 (0.0)	12 (16.0)	28 (9.5)	6 (4.4)	
Have you labelled any patients with drug allergies?						
Yes	126 (69.6)	8 (61.5)	24 (96.0)	68 (69.4)	26 (57.8)	0.028*
In your experience, what are the top 3 most common drug allergies you have labelled?						
β‐lactams	168 (44.4)	8 (33.3)	27 (37.5)	102 (50.0)	31 (39.7)	
Non‐steroidal anti‐inflammatory drugs	55 (14.6)	2 (8.3)	15 (20.8)	20 (9.8)	18 (23.1)	
Radiological contrasts	13 (3.4)	2 (8.3)	5 (6.9)	6 (2.9)	0 (0.0)	
Have you ever encountered patients with anaphylaxis to drugs?						
Yes	94 (51.9)	6 (46.2)	19 (76.0)	42 (42.9)	27 (60.0)	0.054
Mean ± SD	5.41 ± 9.14	8.33 ± 11.08	6.42 ± 11.00	4.86 ± 9.85	4.93 ± 5.92	
Median (min–max)	3 (1–50)	3.5 (1–30)	3 (1–50)	2 (1–50)	3 (1–30)	
Have you ever encountered patients with SCAR to drugs?						
Yes	68 (37.6)	6 (46.2)	21 (84.0)	25 (25.5)	16 (35.6)	< 0.001*
Mean ± SD	6.43 ± 12.56	6.33 ± 6.77	10.29 ± 21.47	4.76 ± 4.49	4.00 ± 3.29	
Median (min–max)	3.5 (1–100)	4 (2–20)	5 (1–100)	3 (1–20)	3 (1–10)	
Is there immediate access to resuscitation facilities and equipment in your current sites of practice (hospital ward and/or clinic)?						
Yes	171 (94.5)	12 (92.3)	24 (96.0)	93 (94.9)	42 (93.3)	1.000
Is there immediate access to intramuscular/subcutaneous adrenaline in your current sites of practice (hospital ward and/or clinic)?						
Yes	171 (94.5)	13 (100.0)	23 (92.0)	92 (93.9)	43 (95.6)	1.000

*Note:* *p < 0.05.

^a^ Computed based on comparison between Advanced Economies (Australia and Hong Kong) and Emerging Economies (Mainland China and Sri Lanka).

Participants from AE reported significantly higher estimated prevalence of DA (*p* < 0.001) in their daily practice compared to those from EE. Specifically, 30.8% (4/13) of Australian and 12.0% (3/25) of Hong Kong participants reported DA prevalence exceeding 10%, compared to only 5.1% (5/98) in Mainland China and 6.7% (3/45) in Sri Lanka. Overall, the most commonly implicated drug classes were BL (41.4%, 225/543), non‐steroidal anti‐inflammatory drugs (NSAID; 19.2%, 104/543) and radiographic contrast agents (8.5%, 46/543). Among the 130 participants (71.8%) who had ever labelled a patient with DA, BL antibiotics were again the leading culprits (43.1%, 168/390 reactions), predominantly penicillins (69.6%, 117/168) and cephalosporins (30.4%, 51/168), followed by NSAID (14.1%, 55/390) and radiological contrasts (3.3%, 13/390).

Participants from AE reported significantly more prior experience managing severe cutaneous adverse reactions (SCAR) than those from EE (71.1% vs. 28.7%, *p* < 0.001). A trend toward greater experience with anaphylaxis was also observed in AE participants, though it did not reach conventional significance (65.8% vs. 48.3%, *p* = 0.054). Nearly all participants (94.5%, 171/181) reported immediate access to resuscitation facilities and intramuscular adrenaline.

**FIGURE 1:**
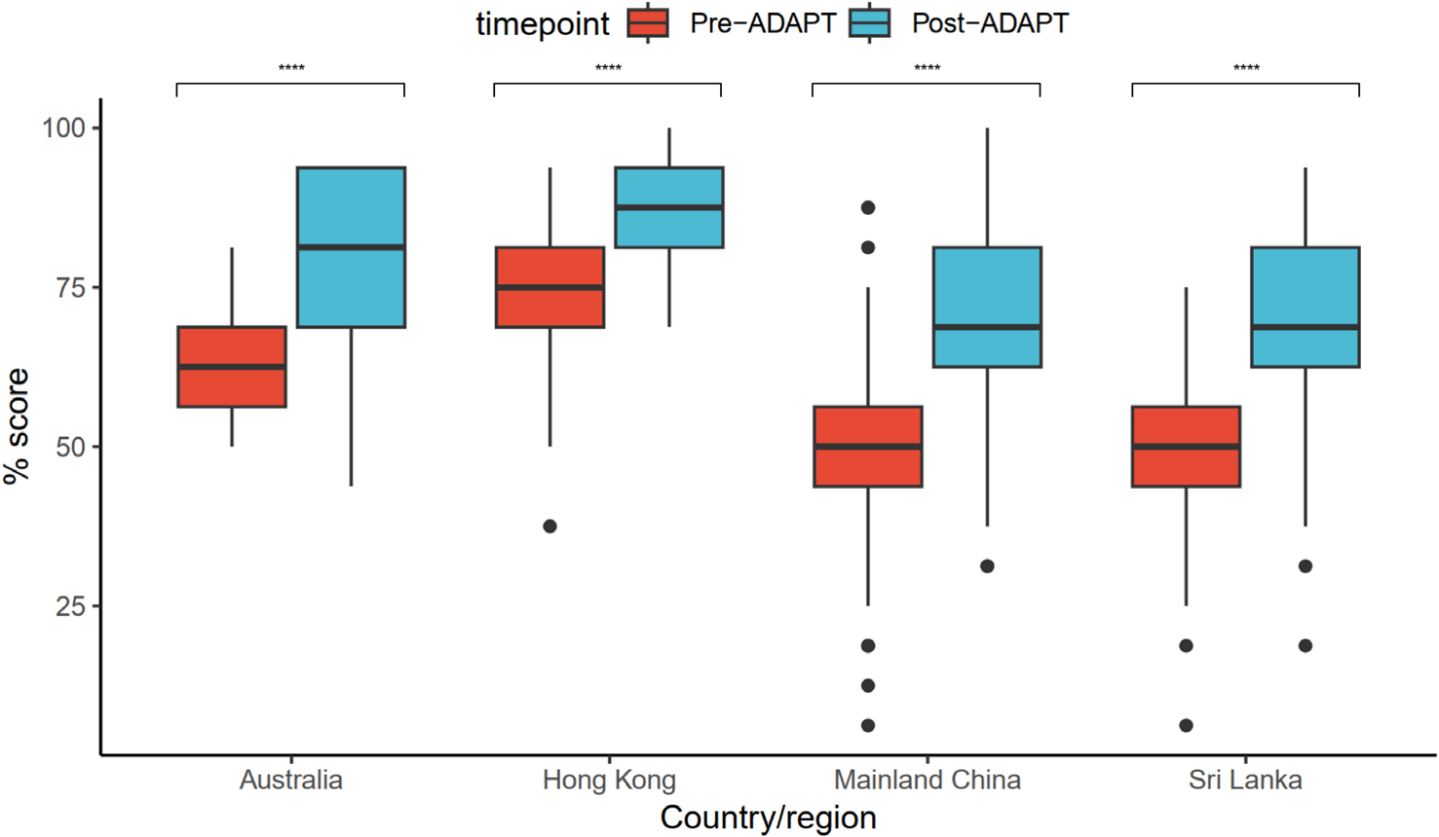
Change in drug allergy knowledge scores following completion of ADAPT.

**FIGURE 2:**
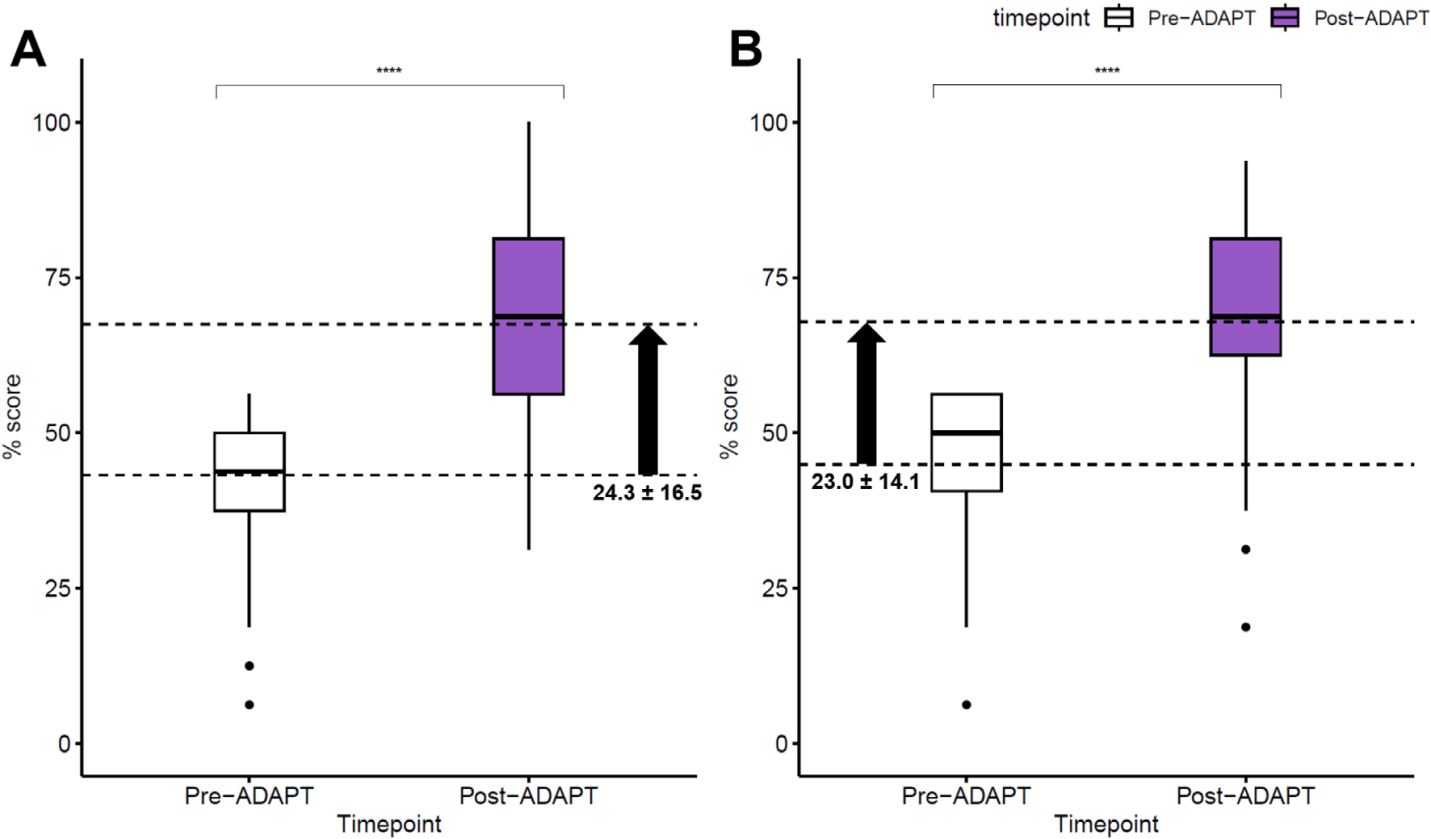
Knowledge change among initially unsatisfactory participants after ADAPT with (A) AI‐assisted video localisation into Chinese and (B) native English.

**FIGURE 3:**
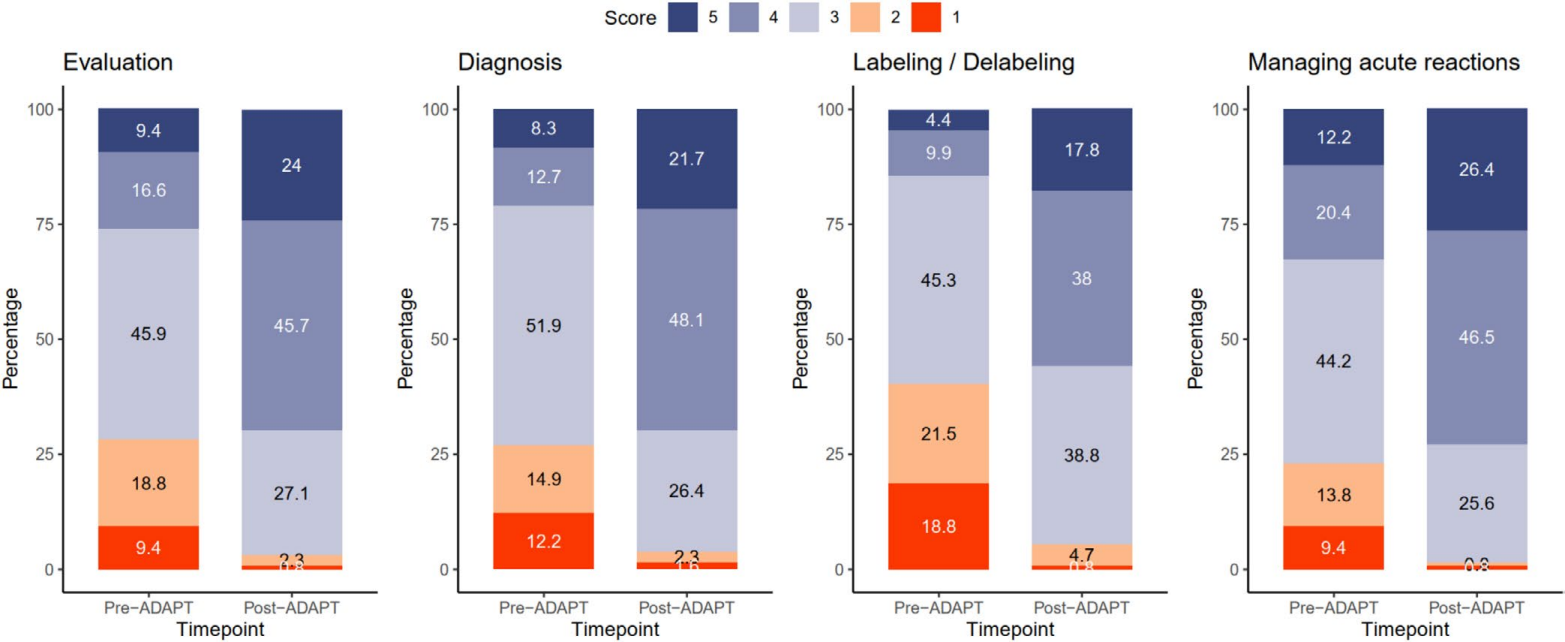
Change in drug allergy confidence scores following completion of ADAPT.

At baseline, the overall knowledge and confidence scores were only 53.5% ± 17.2% and 47.5% ± 22.7%, respectively (Table 3). Of the four surveyed domains (evaluation, diagnosis, labelling/delabelling and managing acute reactions), participants expressed the lowest baseline confidence in labelling/delabeling (mean 2.60 ± 1.04 on a 5‐point Likert scale). Compared to participants from AE, those from EE scored significantly lower in baseline knowledge (49.1% ± 15.5% vs. 70.1% ± 12.7%, *p* < 0.001), but there was no significant difference in confidence (46.8% ± 23.2% vs. 50.2% ± 20.9%, *p* = 0.413). Only 22.4% (32/143) of EE participants were deemed ‘satisfactory’ in DA knowledge, compared to 78.9% (30/38) from AE (*p* < 0.001). Subgroup analysis by profession revealed that internists (knowledge score = 65.8 ± 17.7%) and paediatricians (knowledge score = 65.3% ± 15.7%) scored significantly higher in DA knowledge than family physicians or general practitioners (knowledge score = 54.3% ± 13.3%) and clinicians from other specialties (knowledge score = 50.7% ± 19.1%) (*p* = 0.007; Table S1). Following ADAPT‐2, significant improvements were observed in both knowledge and confidence across all regions. Mean knowledge scores increased from 53.5% ± 17.2% to 72.5% ± 16.0% (*p* < 0.001), and mean confidence scores rose from 47.5% ± 22.7% to 71.3% ± 17.5% (*p* < 0.001) (Figures 1 and 2). The proportion of deemed ‘satisfactory’ in DA knowledge also significantly increased from 34.3% (62/181) to 81.2% (147/181; *p* < 0.001), with all except one AE clinicians (37/38, 97.4%) achieving so. Although confidence in labelling/delabeling remained the lowest among domains, it improved significantly from 2.60 ± 1.04 to 3.67 ± 0.85 (*p* < 0.001). Among the 119 participants who began with unsatisfactory knowledge scores, the AI‐assisted training in Chinese was non‐inferior to the original English course, with a mean improvement of 24.3 ± 16.5 for the AI‐assisted translation, and 23.0 ± 14.1 for the original English, respectively (*p* = 0.646) (Figure 3). Subgroup analyses by economic classification and modality are presented in Figures S1 and S2.

**TABLE 3:** Drug allergy knowledge and confidence scores of participants before (baseline) and after ADAPT.

	Total	Advanced economies		Emerging economies		*p* ^a^
		Australia	Hong Kong	Mainland China	Sri Lanka	
	*N* = 181	*N* = 13	*N* = 25	*N* = 98	*N* = 45	
Baseline						
Knowledge						
Score ≥ 60%	62 (34.3)	8 (61.5)	22 (88.0)	22 (22.4)	10 (22.2)	< 0.001*
Total knowledge score (%)	53.5 ± 17.2	62.5 ± 8.8	74.0 ± 12.7	49.2 ± 15.8	48.9 ± 15.0	< 0.001*
Practice (self‐reported confidence)						
Evaluation (1–5)	2.98 ± 1.05	3.00 ± 0.71	3.12 ± 0.88	2.97 ± 1.15	2.91 ± 1.02	0.432
Diagnosis (1–5)	2.90 ± 1.04	2.77 ± 0.73	3.24 ± 0.93	2.91 ± 1.11	2.73 ± 1.01	0.237
Labelling/delabeling (1–5)	2.60 ± 1.04	2.46 ± 0.88	3.00 ± 1.00	2.69 ± 1.07	2.20 ± 0.94	0.145
Managing acute reactions (1–5)	3.12 ± 1.09	2.85 ± 0.99	3.16 ± 1.18	3.01 ± 1.15	3.42 ± 0.89	0.663
Composite confidence score (%)	47.5 ± 22.7	44.2 ± 14.8	53.3 ± 23.1	47.4 ± 25.2	45.4 ± 18.3	0.413
Post‐ADAPT						
Knowledge						
Score ≥ 60%	147 (81.2)	12 (92.3)	25 (100.0)	75 (76.5)	35 (77.8)	0.004*
Total knowledge score (%)	72.5 ± 16.0	78.4 ± 15.4	86.8 ± 9.8	70.2 ± 15.3	67.9 ± 16.1	< 0.001*
Practice (self‐reported confidence)						
Evaluation (1–5)	3.90 ± 0.82	3.69 ± 0.95	4.16 ± 0.69	3.61 ± 0.91	4.11 ± 0.65	0.368
Diagnosis (1–5)	3.86 ± 0.84	3.54 ± 0.66	4.20 ± 0.65	3.52 ± 0.96	4.11 ± 0.68	0.283
Labelling/delabeling (1–5)	3.67 ± 0.85	3.46 ± 0.88	3.96 ± 0.74	3.50 ± 0.91	3.76 ± 0.80	0.322
Managing acute reactions (1–5)	3.97 ± 0.79	3.77 ± 0.73	4.12 ± 0.67	3.80 ± 0.96	4.11 ± 0.65	0.775
Composite confidence score (%)	71.3 ± 17.5	65.4 ± 17.0	77.8 ± 15.4	65.2 ± 19.5	75.6 ± 14.1	0.346

*Note:* *p < 0.05.

^a^ Computed based on comparison between Advanced Economies (Australia and Hong Kong) and Emerging Economies (Mainland China and Sri Lanka).

**FIGURE 4:**
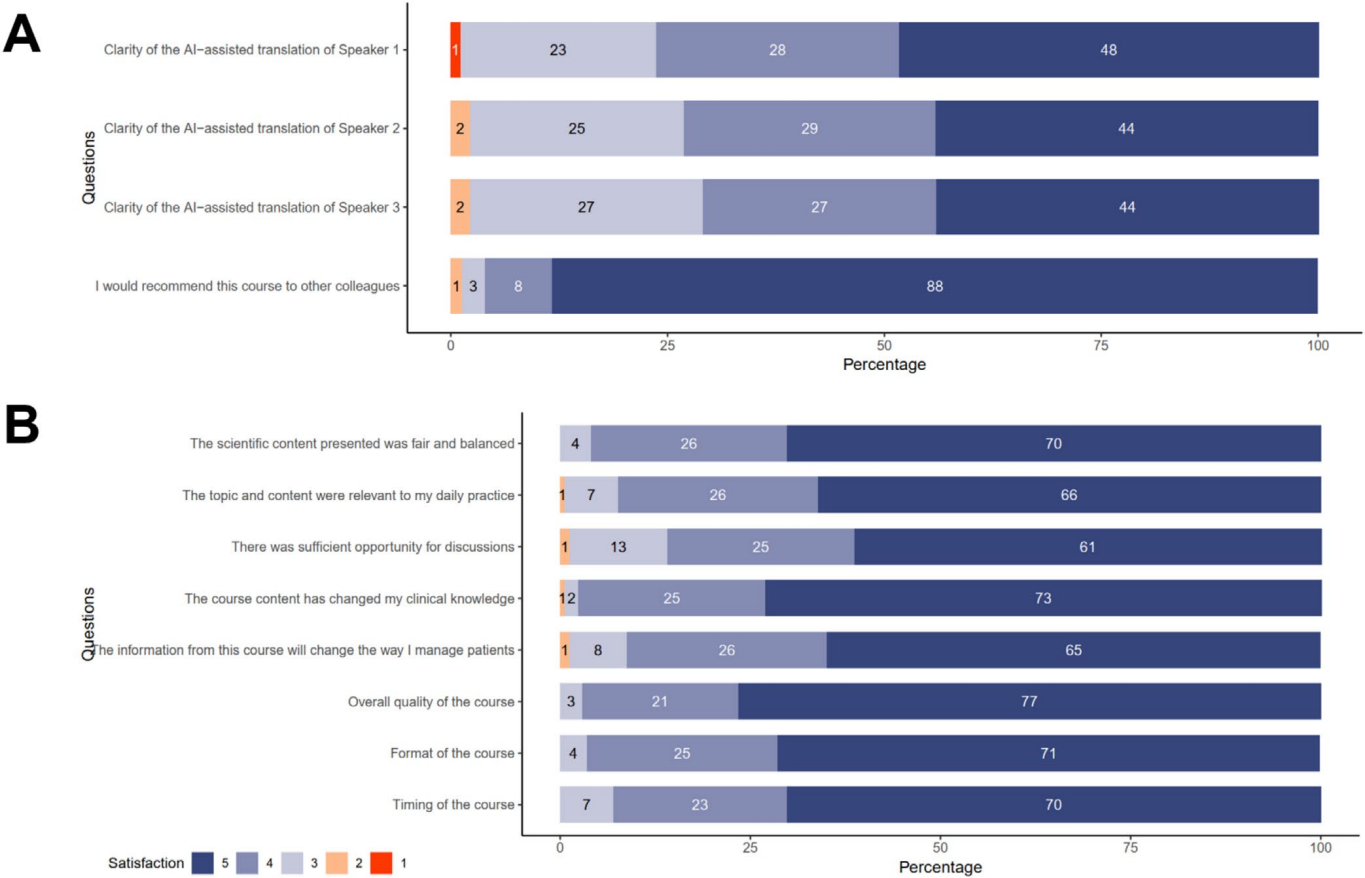
Participant evaluation of (A) AI‐assisted video localisation and (B) ADAPT in general.

Figure 4 shows participants' evaluation of the educational course from 171 (94.5%) participants. Overall, 97.1% (166/171) rated the course quality as 4 or 5 (on a 5‐point scale), and 91.2% (156/171) agreed or strongly agreed that the training would change their clinical management of DA patients. Regarding the AI‐assisted training, nearly all participants in Mainland China rated the clarity and understandability of the content as at least ‘somewhat clear’, with 98.9% (92/93), 97.8% (91/93) and 97.8% (91/93) for the three international faculty speakers whose lectures were translated into Chinese. Nearly half of participants rated the translations as ‘very clear and comparable to native speech’: 48.4% (45/93), 44.1% (41/93) and 44.1% (41/93) for the three speakers, respectively.

Follow‐up outcomes, collected at least 1 month following course completion, were available for 59 participants (32.6%). Sustained improvement in confidence was observed, with no significant difference between post‐course and follow‐up scores (76.0% ± 15.5% vs. 75.5% ± 13.1%, *p* = 0.847). Domain‐specific follow‐up results are detailed in Table S2.

## 4 Discussion

To our best knowledge, this is the first multinational study that evaluates the effectiveness of a structured DA educational course for non‐specialists, and the largest of its kind to date. It is also among the first to investigate the role of AI to assist DA education, with a focus on multilingual content adaptation and scalable implementation across resource‐diverse settings.

At baseline, prior to ADAPT‐2, we observed a marked disparity in baseline DA knowledge between physicians from AE and EE. This gap likely stems from structural, educational and resource‐related barriers, which international, collaborative initiatives like ADAPT aim to address. In many APAC countries, Allergy remains an emerging medical specialty, with limited integration into undergraduate or postgraduate curricula [18, 32, 33, 34]. Structured training pathways, specialist accreditation and dedicated clinical services for DA are often absent [35]. Consistent with this, EE physicians reported significantly less clinical experience with DA, particularly severe manifestations such as anaphylaxis and SCAR, which may reflect both limited exposure and under‐diagnosis of life‐threatening allergic conditions [36]. Notably, many EE participants indicated that ADAPT‐2 was their first formal exposure to DA or Allergy since medical school. This highlights a critical, unmet need for scalable educational programmes to support Allergy capacity building in resource‐constrained regions across the APAC.

Interestingly, despite significantly lower baseline knowledge, physicians from EE reported confidence levels comparable to those from AE. This mismatch between confidence and competence may reflect overconfidence among less knowledgeable clinicians, potentially compromising patient safety through misdiagnosis or inappropriate management [37]. Conversely, the relatively lower confidence observed among some AE physicians may indicate appropriate caution or risk aversion when managing DA without specialist support. Both perspectives highlight the need for urgent intervention to empower non‐specialists to safely and confidently lead or participate in delabeling services, especially for low‐risk patients.

Although DA remains a shared global challenge, effective educational interventions such as ADAPT must be contextually adapted to address region‐specific barriers, particularly where non‐specialists serve as the frontline of care. In several regions of the APAC, including Mainland China, Sri Lanka and India, pre‐emptive and non‐standardised penicillin skin testing remains routine, often performed by clinicians without formal allergy training [32, 35, 38]. This practice leads to widespread mislabelling of penicillin allergy, with false‐positive rates as high as 97% [39]. Consistent with prior literature, our data show that physicians in Mainland China and Sri Lanka assigned significantly more BL allergy labels than counterparts in AE regions such as Hong Kong [8], a pattern likely driven by knowledge gaps and lack of standardised diagnostic pathways [8]. In many EE countries, where DA patients depend almost exclusively on primary health care providers due to limited access to specialist services, empowering non‐specialists through targeted, locally adapted education is essential. Evidence‐based educational interventions such as ADAPT may help bridge this gap by equipping non‐specialists with the knowledge and confidence to safely evaluate, delabel and manage drug allergy.

Moreover, this study further confirms the generalisability and efficacy of structured DA education for non‐specialists. Consistent with the original trial, a single‐day intensive course significantly improved both knowledge and confidence across all participating regions, irrespective of participants' experience or country of practice. While these results affirm that brief, focused interventions can improve DA knowledge, some disparities between AE and EE physicians remained after training. Nearly all AE participants achieved satisfactory knowledge benchmarks after ADAPT‐2, whereas approximately one‐fifth of EE participants did not. This suggests that in settings where Allergy remains an emerging specialty, extended or repeated educational exposure may be necessary.

Complementing these educational gains, our study demonstrates that AI‐translated courses are non‐inferior to their native‐language counterparts in both effectiveness and participant satisfaction. Language barriers have exacerbated disparities to Allergy education between English‐ and non‐English‐speaking regions, particularly in heterogeneous areas like the APAC. Generative AI now represents a novel solution, enabling accurate translation into various languages with near‐native clarity while preserving the original meaning. As in this present study, nearly all participants were satisfied with its clarity and understandability, with almost half of them considering it comparable to native speakers. Nevertheless, human oversight (by bilingual experts with faculty review) remains essential for quality assurance, contextual accuracy and cultural appropriateness. More importantly, use of AI can markedly reduce the time and resources required for content localisation without compromising educational quality. In the future, we plan to leverage this technology to expand ADAPT to additional underserved regions, a critical step toward equitable, evidence‐based allergy care across the APAC.

This study has several limitations. First, confidence metrics were self‐reported and may not fully reflect actual clinical behaviour or decision‐making in practice. Second, we did not evaluate patient‐ or system‐level outcomes, such as delabeling rates, diagnostic accuracy, or changes in antimicrobial prescribing patterns, which would provide stronger evidence of the intervention's real‐world impact. Third, a formal cost‐effectiveness analysis was not conducted. Finally, while one‐month follow‐up data were collected, longer‐term assessments of knowledge retention and sustained practice change are needed, which are currently underway. This study may also be subject to nonresponse bias, where participants with lower follow‐up confidence were less likely to respond. Demographic data of participants from Mainland China was not available.

In conclusion, substantial disparities in DA knowledge and practice persist between physicians from AE and EE. These gaps can be meaningfully reduced through structured, evidence‐based education—as demonstrated by ADAPT‐2. To ensure scalability, sustainability and equity, future efforts should prioritise international collaboration in DA education, with AI‐assisted training serving as a globally scalable tool capable of reaching underserved clinicians, regardless of language or geographic location.

## Author Contributions

H.W.F.M., J.M. and P.H.L. researched the data and wrote the first draft of the manuscript. F.K.L.C., J.T.Y.L., I.S.A., U.A., T.N.D.R., Q.Y., M.Z., L.C., J.‐X.H., W.S., R.K., J.G.P., J.A.T., K.C.J. and M.L. researched the data. M.L. and P.H.L. conceptualised and supervised the study. All authors contributed to and approved the final version of the manuscript before submission.

## Funding

This work was supported by grants to Juan Meng from the National Key Clinical Specialties Fund (no. TJZ202303) and Sichuan Science and Technology Program (no. 2024YFFK0244).

## Conflicts of Interest

Michaela Lucas has received reimbursement for educational talks and conference attendance from CSL, CSL Seqirus, GSK, Stallergenes, Novartis, Boehringer Ingelheim, Sanofi, Arrotec, Menarini and SOBI. All other authors have no conflicts of interest in relation to this work.

## Supporting information


**Table S1:** cea70213‐sup‐0001‐Supinfo.docx.

## Data Availability

The data that support the findings of this study are available from the corresponding author upon reasonable request.
