# Supplementary material for: Bridging Global Disparities in Drug Allergy Through AI‐Assisted Training for Non‐Specialists: Findings From the Multinational ADAPT‐2 Course
Source: Clin Exp Allergy. 2026 Jan 20;56(2):137–46. doi: 10.1111/cea.70213 (PMC12879266; doi:10.1111/cea.70213)

**Supplementary Table S1**. Baseline drug allergy knowledge and confidence scores of participants stratified by demographic and professional background

|  | **N^†^** | **Knowledge** | | **Confidence** | |
| --- | --- | --- | --- | --- | --- |
|  |  | **Total score (%)** | ***P* value** | **Total score (%)** | ***P* value** |
| **Total** | 83 (100.0) | 58.6±17.5 |  | 47.6±19.5 |  |
|  |  |  |  |  |  |
| **Sex** |  |  | 0.973 |  | 0.293 |
| Male | 39 (47.0) | 58.7±18.5 |  | 50.0±18.4 |  |
| Female | 44 (53.0) | 58.5±16.8 |  | 45.5±20.5 |  |
| **Years of clinical experience** |  |  | 0.518 |  | 0.540 |
| <10 years | 42 (50.6) | 59.8±17.7 |  | 46.3±19.0 |  |
| ≥10 years | 41 (49.4) | 57.3±17.4 |  | 48.9±20.2 |  |
| **Type of practice** |  |  | 0.653 |  | 0.386 |
| Private | 23 (27.7) | 59.8±13.0 |  | 44.6±19.5 |  |
| Public | 60 (72.3) | 58.1±19.0 |  | 48.8±19.6 |  |
| **Specialty** |  |  | ***0.007** |  | 0.497 |
| Internal Medicine | 28 (33.7) | 65.8±17.7 |  | 49.6±16.7 |  |
| Family Medicine / General Practitioner | 27 (32.5) | 54.3±13.3 |  | 45.6±18.3 |  |
| Paediatrics | 9 (10.8) | 65.3±15.7 |  | 54.9±31.2 |  |
| Other | 19 (22.9) | 50.7±19.1 |  | 44.1±18.8 |  |

† Data from Mainland China not available

**Supplementary Table S2**. Follow-up outcomes (N = 59)

|  | **Pre-ADAPT** | **Follow-up** | ***P* value** | **Post-ADAPT** | **Follow-up** | ***P* value** |
| --- | --- | --- | --- | --- | --- | --- |
| ***Practice (self-reported confidence)*** |  |  |  |  |  |  |
| Evaluation (1–5) | 2.93±0.96 | 4.03±0.69 | ***<0.001** | 4.12±0.70 | 4.03±0.69 | 0.480 |
| Diagnosis (1–5) | 2.79±1.00 | 4.10±0.61 | ***<0.001** | 4.15±0.72 | 4.10±0.61 | 0.635 |
| Labelling / Delabeling (1–5) | 2.30±0.99 | 3.75±0.69 | ***<0.001** | 3.80±0.85 | 3.75±0.69 | 0.684 |
| Managing acute reactions (1–5) | 3.21±1.07 | 4.20±0.58 | ***<0.001** | 4.08±0.68 | 4.20±0.58 | 0.211 |
| Composite confidence score (%) | 45.2±19.4 | 75.5±13.1 | ***<0.001** | 76.0±15.5 | 75.5±13.1 | 0.847 |

**Supplementary Figure S1**. Confidence change after course among participants from (A) advanced economies (B) emerging economies


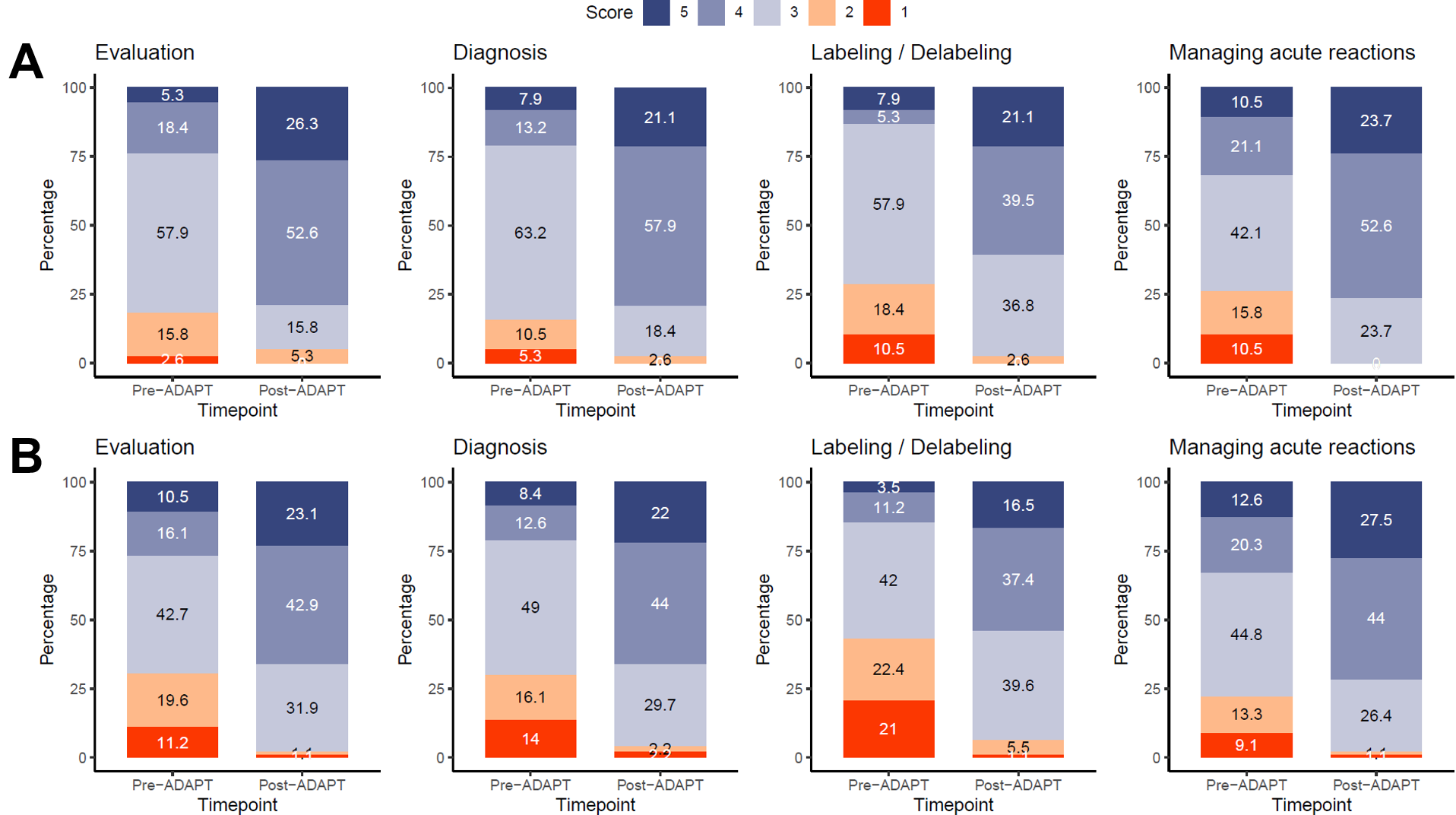


**Supplementary Figure S2**. Confidence change after ADAPT with (A) AI-assisted video localization into Chinese and (B) native English


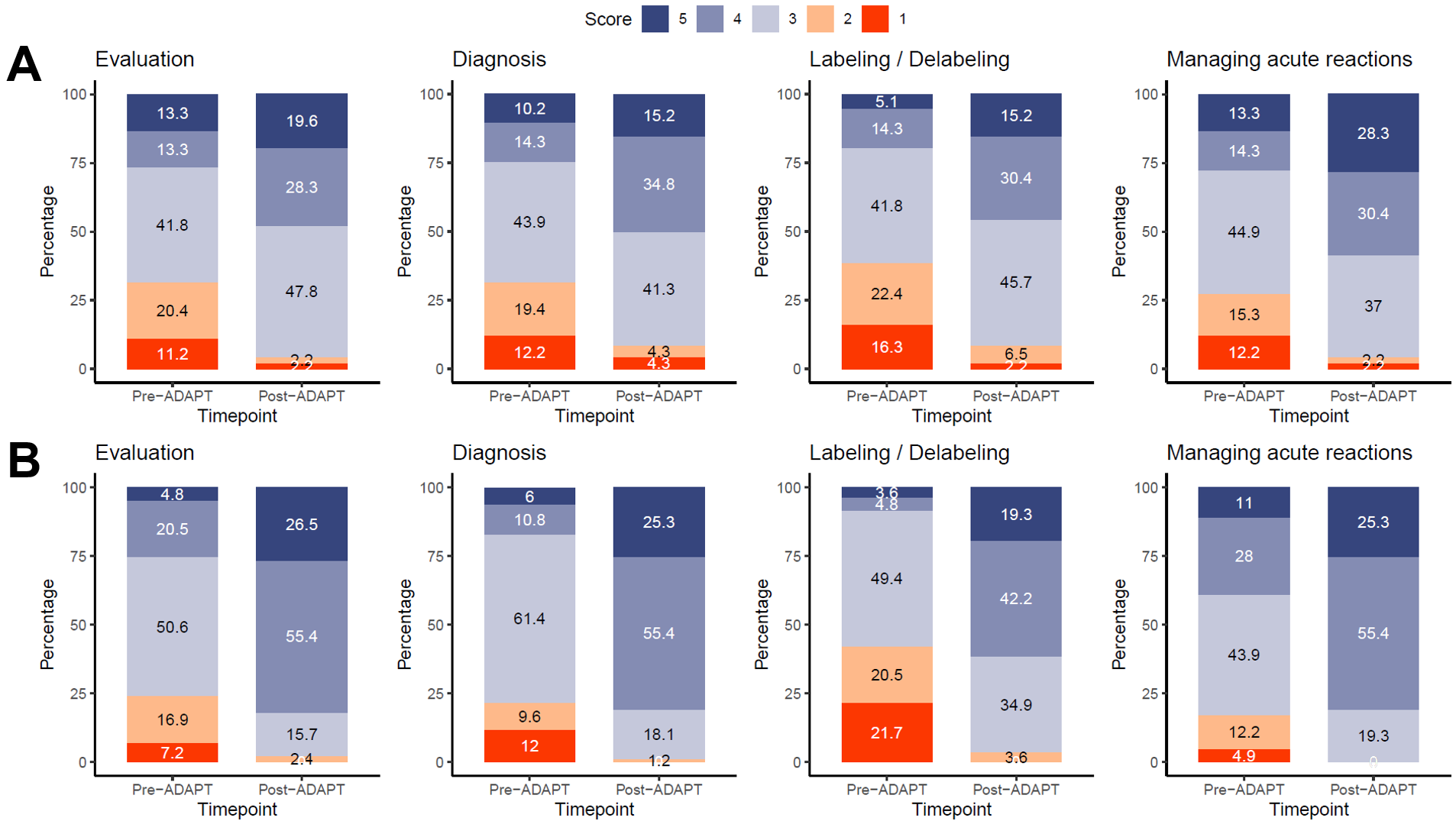

Supplement: Supplementary file 1 — Table S1: cea70213‐sup‐0001‐Supinfo.docx. [file CEA-56-137-s001.docx]
